# Complexities of *Candida* Colonization and Oral Microbiome in Oral Lichen Planus: A Systematic Review and Meta-Analysis

**DOI:** 10.3390/dj13070310

**Published:** 2025-07-10

**Authors:** Israyani, Christine Anastasia Rovani, Erni Marlina, Chung-Ming Liu

**Affiliations:** 1 Graduate Institute of Dental Science, College of Dentistry, China Medical University, Taichung 404, Taiwan; u111312101@cmu.edu.tw (I.); u111312102@cmu.edu.tw (C.A.R.); 2 Oral Medicine Department, Faculty of Dentistry, Hasanuddin University, Makassar 90245, Indonesia; erni.marlina@unhas.ac.id; 3 Department of Oral Medicine, Dental Hospital of Hasanuddin University, Makassar 90156, Indonesia; 4 Conservative Dentistry Department, Faculty of Dentistry, Hasanuddin University, Makassar 90245, Indonesia; 5 Department of Biomedical Engineering, College of Biomedical Engineering, China Medical University, Taichung 404, Taiwan; 6 Department of Dentistry, Health Sciences University of Hokkaido, Tobetsu 061-0293, Japan

**Keywords:** oral lichen planus, oral microbiome, microbiota, fungal colonization

## Abstract

**Background/objectives:** Oral lichen planus (OLP) is a chronic autoimmune disorder affecting various age groups and is associated with multiple factors. Conventional therapies often encounter complications from opportunistic infections, particularly oral candidiasis. This study examines the relationships between *Candida* colonization and oral microbiome composition in OLP patients. Through meta-analysis, we clarify these interactions and their implications for OLP progression. **Methods:** The PICOS is a systematic research strategy, following PRISMA 2020 and MeSH descriptors: oral lichen planus, oral microbiome, oral fungal, and non-*Candida* oral fungal. **Results:** A search of CINAHL, EMBASE, PubMed, Science Direct, and Web of Science identified 313 studies. Twelve studies were suitable for a systematic review, with four appropriate for meta-analysis. Findings showed a significant association between OLP and oral microbiota, with an OR of 4.155 (95% CI: 1.278–13.511, *p* = 0.024). Although analyses of *C. albicans* and non-*albicans* species lacked significance, particular non-*albicans* species were noted. The subgroup analysis of oral microbiota approached significance, indicated by an OR of 11.739 (95% CI: 0.654–210.713, *p* = 0.059). **Conclusions:** This study highlights the roles of *Candida* species and the oral microbiota in OLP, revealing a complex interaction between *Candida* colonization and the oral microbiome.

## 1. Introduction

Oral lichen planus (OLP) is a relatively common chronic inflammatory disease affecting the oral mucosa, with a global prevalence ranging from 1% to 2% [1,2,3]. This autoimmune disorder is characterized by the activation of cytotoxic T-lymphocytes, which trigger the apoptosis of epithelial cells and result in persistent inflammation within the oral cavity [1,4]. Clinically, OLP presents with various lesions, including reticular, erosive, atrophic, and plaque-like forms [4,5]. These cause pain, significantly impacting a patient’s quality of life. Diagnosis relies on clinical examination and histopathological confirmation. OLP may mimic other oral diseases and is linked to a higher risk of malignant transformation [6,7,8,9].

The pathogenesis of OLP is complex and multifactorial, involving genetic predisposition, environmental factors, and immune dysregulation [10,11,12]. T-lymphocytes play a central role in the disease process, infiltrating the epithelium and lamina propria, releasing pro-inflammatory cytokines, and contributing to chronic inflammation [13,14]. Additionally, alterations in the oral microbiota may be associated with susceptibility to fungal infections.

The oral microbiome, a diverse community of microorganisms inhabiting the oral cavity, is critical in maintaining oral health [15,16]. This ecosystem comprises bacteria, fungi, viruses, and archaea that interact with each other and the host immune system. Dysbiosis, an imbalance in the oral microbiome, is linked to various oral diseases, including dental caries, periodontal disease, and OLP [17,18].

*Candida* species, a group of opportunistic fungi, are common inhabitants of the oral cavity [19,20]. However, under certain conditions, such as immune suppression or disruption of the oral microbiome, *Candida* can proliferate and cause oral candidiasis, also known as thrush [21,22,23]. The connection between *Candida* and OLP remains under investigation. Studies indicate that *Candida* colonization may worsen OLP lesions or contribute to their pathogenesis [24,25]. However, the specific roles of *Candida* species and the broader oral mycobiome in OLP remain unclear [24,26,27,28].

Although previous reviews have primarily focused on *Candida albicans* in OLP, most notably the meta-analysis by Rodriguez-Archilla and Fernandez-Torralbo (2022) [18], these studies have not systematically addressed the roles of non-albicans *Candida* species or the broader mycobiome. Recent evidence suggests that fungal dysbiosis may extend beyond *C. albicans*, with non-albicans species and other fungal genera playing contributory roles in the immunopathogenesis of OLP. Our study seeks to fill this gap by not only examining *C. albicans* but also analyzing non-albicans species and overall mycobiome profiles in OLP patients using both traditional and sequencing-based data. This expanded scope allows for a more comprehensive understanding of fungal contributions to OLP and represents a novel addition to the current body of literature.

The oral microbiome plays a crucial role in maintaining oral ecosystem balance [29,30]. Modifications in oral microorganism composition have been linked to various diseases, including OLP [31,32]. This study highlights these roles and the oral microbiota in OLP, revealing a complex interaction between fungal colonization and the oral microbiome [33]. Research suggests fungal infections alter oral microbiota composition and worsen symptoms; conversely, changes may increase fungal susceptibility to infections [34,35].

This systematic review and meta-analysis aimed to shed light on the roles of fungal infections and the oral microbiome in the pathogenesis of OLP. The objectives included assessing the relationship between *Candida* colonization (both *Candida* and non-*Candida*) and OLP, investigating the oral microbiome’s impact on OLP pathogenesis, and proposing prevention and treatment strategies. The findings of this study could significantly enhance the understanding of OLP pathogenesis and help guide targeted therapeutic interventions. Specifically, this review focuses on the main research question: how does *Candida* colonization and/or oral microbiome dysbiosis vary in patients with oral lichen planus compared to healthy individuals?

## 2. Method

### 2.1. Search Strategy

This study registered under CRD42024604254 in Prospero on 1 November 2024, explored the impact of OLP on the oral fungal microbiome compared to healthy controls. It focused on patients with OLP and profiling their oral microbiomes, with healthy individuals as the comparator group. The primary outcome was the differences in the composition of the oral fungal microbiome between these groups. A comparative cross-sectional design was employed, guided by the Population, Intervention, Comparator, Outcome, and Study Design (PICOS) framework. The literature was applied using Boolean operators “AND” and “OR” with search terms including oral lichen planus, oral microbiome, oral fungal, and non-Candida oral fungal. The literature search included studies published until 11 December 2024, across databases such as CINAHL, EMBASE, PubMed, Science Direct, and Web of Science. Only original research articles were considered, adhering to PRISMA guidelines [36].

### 2.2. Study Selection Criteria

**Figure 1:**
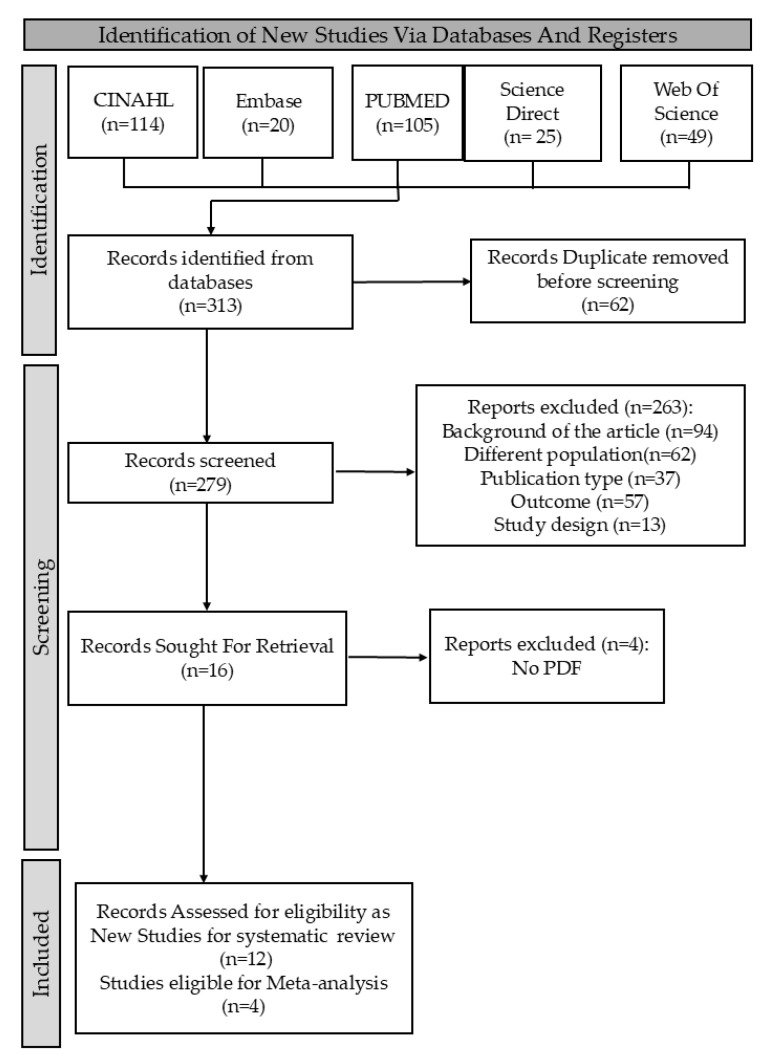
PRISMA flowchart summarizing the systematic review process.

In this research, I. and C.A.R. searched databases for relevant papers using specific terms, with C.-M.L. confirming findings. We retrieved 313 studies, screened for eligibility based on inclusion and exclusion criteria, and used the new Rayyan website to remove 62 duplicates. We ultimately focused on studies meeting the PICOS criteria: (1) diagnosis of OLP; (2) analysis of oral microbiome; (3) outcomes showing differences in oral fungal microbiome; and (4) inclusion of both Candida and non-Candida fungi. Exclusion criteria were (1) inappropriate interventions based solely on conventional cultures; (2) studies lacking specific cancer outcomes; and (3) studies not comparing OLP patients with healthy individuals. Only studies that met all PICOS criteria and used clearly defined diagnostic and microbiological methods were included. This process is detailed in the PRISMA flowchart (Figure 1).

### 2.3. Data Extraction

Two reviewers independently extracted data using a standardized form. The information extracted from each study included the following: first author, publication year, title, study design, sample size, patient demographics (age, gender, ethnicity), diagnostic criteria for OLP, methods of microbial identification, identified Candida species, prevalence of Candida and other fungal species in OLP patients and controls, and main findings related to oral microbiome composition. Discrepancies in data extraction were resolved through a discussion consensus.

### 2.4. Quality Assessment

In this study, we employed the ROBINS-I (Risk Of Bias In Non-randomized Studies—of Interventions) tool to assess the potential risk of bias across seven domains critically. For each included study, two reviewers independently applied the ROBINS-I signaling questions to evaluate (1) bias due to confounding—by examining whether studies were adjusted for key prognostic variables such as age, gender, and corticosteroid use; (2) bias in selection of participants—by assessing how OLP patients and healthy controls were recruited and whether inclusion criteria were clearly defined and consistently applied; (3) bias in classification of interventions—by determining the accuracy and consistency of microbial identification methods (e.g., culture-based vs. sequencing); (4) bias due to deviations from intended interventions; (5) bias due to missing data; (6) bias in measurement of outcomes—by evaluating the objectivity and blinding of microbiological assessments; and (7) bias in selection of the reported result. Any disagreements were resolved through consensus with a third reviewer (C.-M.L.). This structured approach enabled us to assign a final judgment (low, moderate, serious, or critical risk) for each domain and overall per study [37,38].

### 2.5. Statistical Analysis

We opted for a random-effects model to accommodate the inherent variability among the studies in our analysis. This methodology provides a comprehensive framework for interpreting the observed outcomes, facilitating a deeper and more generalized understanding of the phenomena we are examining. Specifically, we utilized the Mantel–Haenszel (MH) pooling method, which is particularly well-suited for the analysis of binary data [39,40]. By employing this method, we aim to enhance the reliability of our findings while offering a more precise and comprehensive interpretation of the data. All statistical analyses were two-tailed, and *p*-values were reported with exact values without spacing (e.g., “*p* = 0.024”), by standard reporting guidelines. All statistical analyses and meta-analytic computations were performed using R software (version R 4.3.2; R Foundation for Statistical Computing, Vienna, Austria) with the meta 6.5-0 for implementing Mantel–Haenszel random-effects models, forest plots, and funnel plot generation.

## 3. Result

### 3.1. Study Selection Result

This review identified 313 records from five databases. We screened the literature, removing 62 duplicate studies for uniqueness. Next, we assessed 279 titles and abstracts for relevance, excluding 263 studies that did not meet criteria. This left 16 articles for eligibility assessment, and after evaluation, we excluded 4 more based on criteria, resulting in 12 studies for our analysis. This systematic approach streamlined selection and enhanced our findings, as shown in Figure 1.

### 3.2. Study Characteristics

Table 1 summarizes the characteristics of the 12 included studies. The studies were published between 2011 and 2024 and conducted in various countries, including China, Denmark, Italy, Germany, India, Japan, and Iran. The sample sizes ranged from 5 to 268 for the OLP group and 0 to 25 for the control group. The age range of participants, spanning from 14 to 88 years, coupled with diverse gender distributions, underscores the need for standardized demographic reporting to facilitate comparative analyses [33,41,42,43,44,45,46,47,48,49,50,51].

### 3.3. Overall Findings

**Figure 2:**
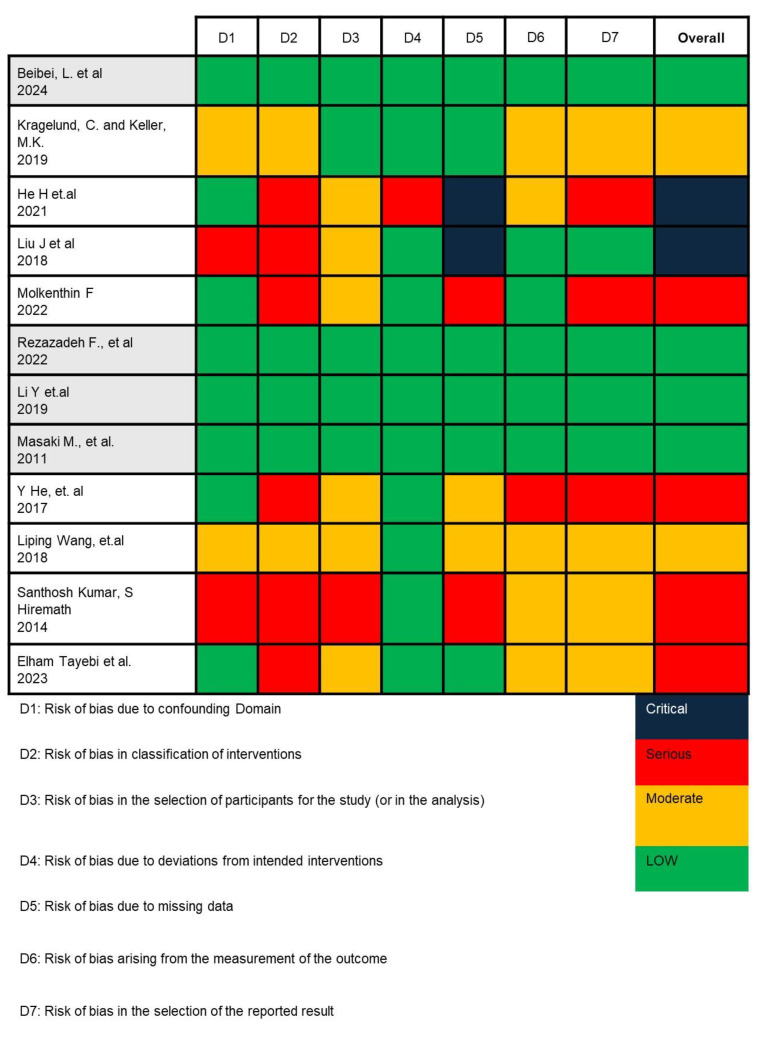
Risk of bias analysis result using ROBINS-I V2 [33,41,42,43,44,45,46,47,48,49,50,51].

Based on the risk of bias analysis shown in Figure 2, conducted by two researchers (I. and C.A.R.) and confirmed by the team leader (C.-M.L.), it was found that the research by Beibei, L. et al., 2024; Rezazadeh F. et al., 2022; Li Y et al., 2019; and Masaki M. et al., 2011 is appropriate to continue in the meta-analysis [41,45,46,47].

Table 2 presents the results from four studies examining the presence of *Candida* species in patients with OLP compared to control groups, indicating a generally low risk of bias. Sample sizes for OLP groups ranged from 15 to 49, and controls from 7 to 32. *Candida albicans* was notably absent in Beibei’s study but was prevalent in other OLP groups. Non-*albicans Candida* species showed limited presence in OLP and control groups, including *C. glabrata*, *C. fukuyamaensis*, *C. paraphimosis*, and various other genera like *Aspergillus* and *Talaromyces*. Detailed analysis of oral microbiomes revealed diverse fungal species in OLP groups, underscoring a complex relationship between OLP and oral fungal microbiota.

### 3.4. Odds Ratio Analysis

This meta-analysis examines the relationship between OLP patients and control groups by analyzing data from multiple studies categorized into three main groups: *Candida albicans*, non-*albicans Candida*, and the oral microbiome. To account for the variations among these studies, we employed a random-effects model and the Mantel–Haenszel (MH) pooling method, which is particularly effective for binary data. Additionally, we evaluated variability using the Paule–Mandel (PM) method. Effect sizes were determined by calculating odds ratios (ORs) for each study, subgroup, and the overall data set. Furthermore, we utilized I^2^, tau^2^, and Chi^2^ statistics to examine heterogeneity and assess the variability of the studies. Although I^2^ values were consistently 0% across subgroups and the overall analysis, this likely reflects the small number of studies included per subgroup and limited statistical power to detect between-study variability. To ensure comparability, subgroup definitions were standardized as follows: (1) all included studies diagnosed OLP based on clinical and histopathological criteria; (2) fungal identification techniques were categorized by type—culture-based or next-generation sequencing (NGS); and (3) subgroup analysis was conducted separately for *C. albicans*, non-albicans *Candida*, and total oral microbiota profiles. This stratification minimized methodological heterogeneity and justified the pooling of studies within each subgroup. Only four studies met the strict inclusion criteria for meta-analysis due to the requirement for consistent fungal species identification and availability of raw dichotomous outcome data. While this limited the size of the quantitative synthesis, it improved methodological rigor and reduced heterogeneity.

**Figure 3:**
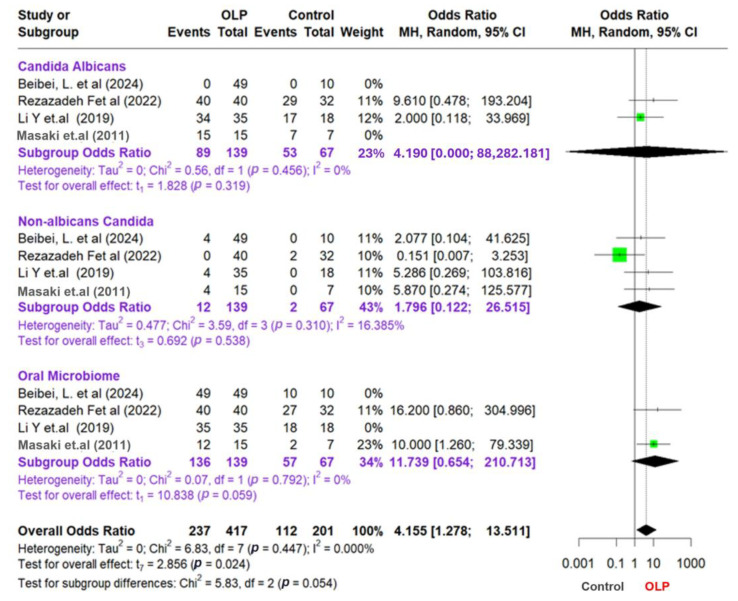
Forest plot and funnel plot assessing the association between oral lichen planus and microbial factors [41,45,46,47].

The study findings are presented in a forest plot shown in Figure 3, which allows for a clear comparison of odds ratios (ORs) across different studies and subgroups. To assess potential publication bias, we also created a funnel plot that reveals effect sizes relating to sample sizes or standard errors, helping to identify any asymmetrical patterns that may indicate bias or variations among the studies. Combining these visual tools, we examine the statistical relationships among subgroups, emphasizing the importance of valid and reliable results in our meta-analysis. We aim to offer insightful perspectives on the complex interactions within the microbiome and related conditions. To supplement the visual assessment of publication bias via funnel plots, we applied Egger’s regression asymmetry test using the log odds ratio standard error as a predictor. However, given that only four studies were included in the meta-analysis, we recognize the limited power of graphical and statistical methods to detect asymmetry. The trim-and-fill method was not used, as it is generally not recommended when fewer than ten studies are available due to instability in imputations.

This study of meta-analysis provides information on the relationship between OLP, *Candida*, and the oral microbiota. The result shows that the subgroup analysis focusing on *Candida albicans* yielded an odds ratio (OR) of 4.19; however, this result was not statistically significant (*p* = 0.319). The low level of heterogeneity observed in this subgroup (I^2^ = 0%) suggests a uniformity across the studies examined. Conversely, the non-*albicans Candida* subgroup also presented insignificant findings, with an OR of 1.796 (95% CI: 0.122–26.515; *p* = 0.538) and a low heterogeneity level (I^2^ = 16.385%). These results emphasize the need for further research to clarify the role of *Candida* in OLP and its implications for oral health.

Furthermore, in the oral microbiota subgroup, the OR value was 11.739 (95% CI: 0.654–210.713), which approached statistical significance (*p* = 0.059) and exhibited a very low level of heterogeneity (I^2^ = 0%). The overall analysis results showed an OR of 4155 (95% CI: 1278–13,511), which was statistically significant (*p* = 0.024). This indicates a meaningful relationship between OLP and the oral microbiota. The heterogeneity between studies in the overall analysis was also low (I^2^ = 0I^2^ = 0%I^2^ = 0%), showing consistent results across different studies.

The test of the difference between the subgroups produced a *p*-value of 0.054, which indicates a difference between the subgroups, although it is not statistically significant. However, the analysis at the subgroup level has insignificant results, which can be due to the limited amount of data or sample size in each subgroup.

This meta-analysis also included studies where one or both groups had zero events, particularly in the *Candida albicans* and non-*albicans* subgroups. A standard continuity correction of 0.5 was applied to all four cells of the 2 × 2 table to enable OR estimation in these cases. This correction prevents division by zero and facilitates the inclusion of all relevant studies. The Mantel–Haenszel random-effects model was used, as it is appropriate for binary outcomes with rare events. We did not apply the Peto method because it may introduce bias in situations involving unbalanced sample sizes or moderate to large treatment effects.

**Figure 4:**
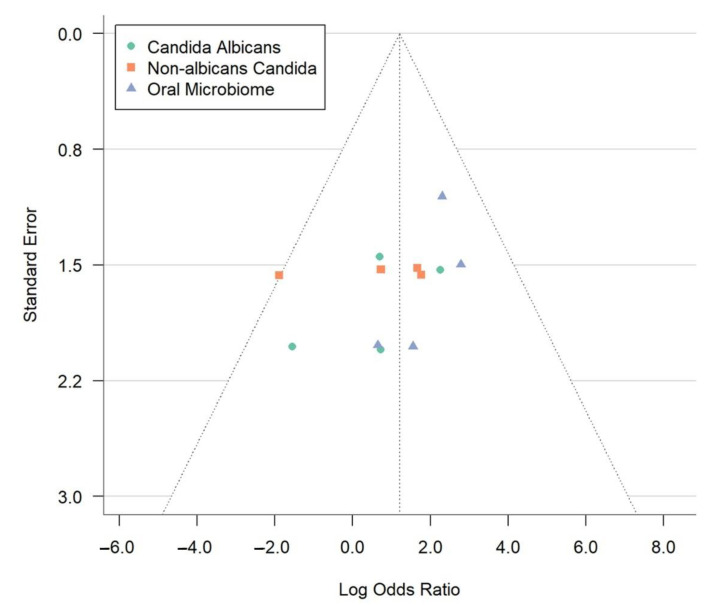
Funnel plot evaluating publication bias in meta-analysis of oral lichen planus and microbial associations.

The image depicts a funnel plot (Figure 4), a crucial instrument in meta-analyses that helps identify publication bias and study heterogeneity. This plot shows how each study’s standard error (y-axis) relates to the log odds ratio (x-axis). Each point represents a study, grouped into three categories: *Candida albicans* (green circles), non-*albicans Candida* (orange squares), and the oral microbiome (blue triangles). A lower standard error means more precise estimates. The log odds ratio measures the link between OLP and microbial factors. The vertical line at a log odds ratio of 2.0 represents the overall effect size from the meta-analysis.

In an ideal funnel plot without bias, data points form a symmetrical funnel shape around the combined effect size, indicating minimal publication bias. The dotted lines create a triangle, showing the expected study distribution. Accurate studies cluster at the top, while less precise ones spread towards the bottom. The observed symmetry suggests a slight selective publication bias in the meta-analysis, enhancing the credibility. Nonetheless, given the limited number of studies (*n* = 4), we recognize that both the funnel plot and Egger’s test have low sensitivity in detecting true publication bias, and findings should be interpreted cautiously. The data points analyze microbial factors, revealing reliable findings for *C. albicans* and the oral microbiome. Minor variations do not affect the symmetrical distribution, supporting the meta-analysis conclusions. This visual analysis highlights the role of microbial factors in OLP development, emphasizing the need for ongoing research for effective treatment strategies.

To better contextualize the novelty of our findings, we compared the effect sizes and heterogeneity metrics reported in previous works with those derived from our current meta-analysis (Table 3). Most prior studies, such as those by Bankvall et al. (2023) [31] relied on sequencing-based analyses without quantifying pooled effect sizes or evaluating heterogeneity. The only comparable meta-analysis (Rodriguez-Archilla & Fernandez-Torralbo, 2022) [18] was limited to *C. albicans* and yielded a lower pooled OR with modest heterogeneity. In contrast, our meta-analysis incorporates broader fungal diversity, including non-albicans species and microbiome shifts, and shows stronger associations with minimal heterogeneity. These comparisons underscore the enhanced analytical scope and robustness of our findings.

## 4. Discussion

This review and meta-analysis examined the relationship between OLP and the oral fungal microbiome, focusing on *Candida* species. Our findings indicate a significant association between OLP and Candida colonization in the oral cavity; however, given the cross-sectional and observational nature of the included studies, no causal inference can be made. Individuals with OLP generally have a higher prevalence of *Candida* than healthy individuals, aligning with previous studies reporting increased *Candida* in OLP lesions. *Candida albicans* emerged as the most frequently linked species to OLP, reinforcing its role as an opportunistic pathogen in the oral environment. Other *Candida* species were also found, but their tie to OLP was less prominent [47,53].

Several factors may explain the observed association between OLP and *Candida*. First, OLP lesions often exhibit epithelial disruption and ulceration, creating a favorable environment for *Candida* colonization. Second, the altered immune response in OLP patients may impair their ability to control *Candida* growth effectively. Third, medications commonly used to manage OLP, such as corticosteroids, can increase the risk of *Candida* infections.

Our analysis explored the role of the non-*Candida* fungal microbiome in OLP. Although data were limited, OLP patients may have different non-*Candida* fungal species compared to healthy individuals [53,54]. This implies that the fungal community, not just *Candida*, may affect OLP pathogenesis. Further research is needed to identify the specific non-*Candida* species and their mechanisms. The oral microbiome maintains oral balance, and changes in microorganism composition are linked to various diseases, including OLP. Research indicates fungal infections alter oral microbiota, worsening symptoms, while microbial changes may also affect susceptibility to fungal infections [52,53,54,55]. A similar result was found in an earlier published meta-analysis focusing on the oral microbiome composition in OLP patients, which also reported a significant difference but could not identify specific microbial taxa driving this association. Li et al. (2019) and Imabayashi et al. (2016) show that patients with OLP have a distinct oral microbiome profile compared to healthy individuals [46,52]. In contrast to previous studies by Swidergall & Filler (2017), He et al. (2017), Masaki et al. (2010), and Li et al. (2019) show a statistically significant association between OLP and specific Candida species [22,46,47,48].

This review’s strengths include its comprehensive search strategy, established meta-analytic methods, and the inclusion of a diverse range of studies examining both *Candida* species and the broader oral microbiome [23,56,57]. The association between OLP and oral microbiota (OR = 11.739, 95% CI: 0.654–210.713, *p* = 0.059) approached statistical significance, suggesting a potential involvement of other microbial factors besides *Candida* in OLP pathogenesis. However, given the small number of studies and limited sample size, this finding should be interpreted cautiously and validated in future research with larger cohorts. The microbiome analysis, revealing diverse fungal species in OLP groups, supports this. Further research on these species and their interactions is essential for understanding the complex relationship between the oral mycobiome and OLP.

These findings have important implications for the clinical progression of OLP. Although causality cannot be inferred, the elevated prevalence of *Candida* species, particularly in erosive forms of OLP, suggests that fungal colonization may act as a cofactor that exacerbates mucosal damage, prolongs inflammation, or complicates lesion healing [18,20]. Patients receiving immunosuppressive agents (e.g., corticosteroids) may be particularly vulnerable to opportunistic fungal overgrowth, which could further shift the microbial balance toward dysbiosis [58]. Moreover, non-*albicans Candida* species, often less responsive to conventional antifungals, may indicate evolving patterns of resistance or deeper mucosal involvement [59]. Recognizing these microbial patterns could support risk stratification and prompt consideration of adjunctive antifungal therapy to mitigate symptom severity, reduce recurrence, or prevent secondary infections.

Clinical subtype appears to be an essential mediator of microbial shifts in OLP. Erosive and atrophic forms of OLP, characterized by ulceration and epithelial thinning, create a permissive environment for *Candida* colonization and broader mycobiome disruption [20,32]. These subtypes are more painful and symptomatic and may exhibit more pronounced immune dysregulation and microbial imbalance. In contrast, reticular or plaque-like OLP often maintain a more intact mucosal barrier, which may be less susceptible to colonization. Several studies in this review observed higher rates of Candida isolation or fungal diversity in erosive than non-erosive lesions [60,61]. This interaction suggests that microbial colonization may reflect disease severity and contribute to symptom persistence and resistance to therapy [18,62]. Future research should stratify participants by clinical subtype to better understand the bidirectional relationship between mucosal integrity and microbial ecology in OLP.

**Figure 5:**
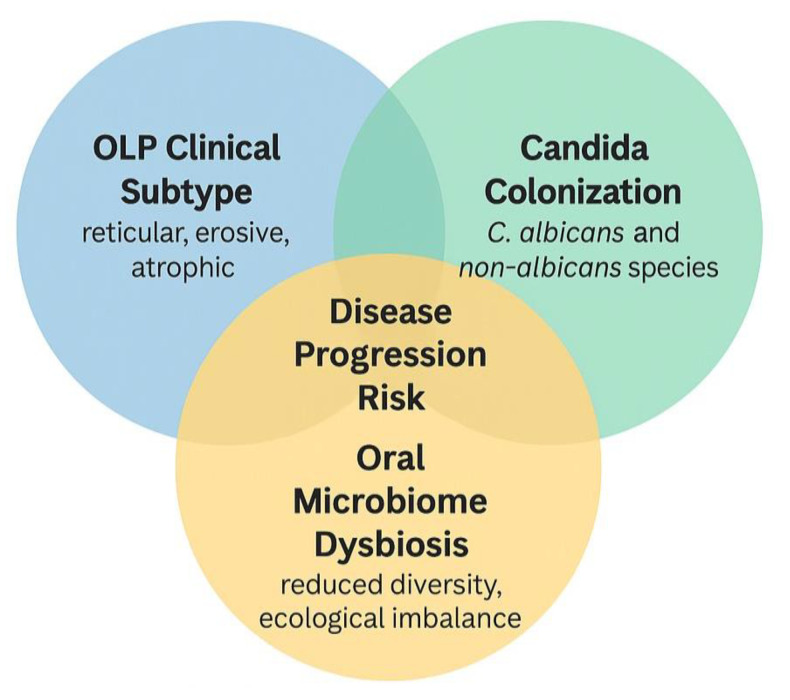
Proposed interaction between oral lichen planus clinical subtype, *Candida* colonization, oral microbiome dysbiosis, and disease progression.

Figure 5 shows the synthesizes of the interaction between oral lichen planus (OLP) clinical subtypes, *Candida* colonization, and oral microbiome dysbiosis, and their potential collective contribution to disease progression. Erosive and atrophic OLP subtypes are particularly vulnerable to microbial colonization due to epithelial barrier disruption. *Candida* species (both *C. albicans* and non-*albicans*) may exacerbate inflammation in this context. Concurrent oral mycobiome dysbiosis, such as reduced diversity and dominance of pathogenic fungi, may further aggravate immune imbalance. Their intersection is proposed to increase the risk of clinical worsening, symptom persistence, and treatment resistance. The overlapping region identifies individuals at higher risk for adverse clinical outcomes, where targeted screening and adjunctive antifungal strategies may be especially beneficial.

## 5. Conclusions

This systematic review and meta-analysis revealed a significant association between oral lichen planus (OLP) and oral fungal colonization, particularly involving *Candida albicans*, with emerging evidence suggesting the potential relevance of non-*albicans* species and broader mycobiome dysbiosis. Although causality cannot be inferred due to the observational nature of the included studies, the findings suggest that fungal imbalance, especially in erosive OLP subtypes, may reflect or exacerbate mucosal disruption. Clinicians should consider fungal screening in OLP patients, particularly when symptoms persist despite conventional treatment or when corticosteroids are used. Future studies with standardized reporting of clinical subtypes, fungal profiles, and longitudinal data are needed to further elucidate fungal communities’ role in OLP progression and treatment responsiveness.

## Figures and Tables

**Table 1:** Characteristic studies included in the systematic review.

First Author	Region	Study Design	OLP	Control	Age of Participant	Population Sex
Beibei 2024 [41]	China	Observational, cross-sectional, controlled, single-site	49	10	Control: 45.6 ± 10.9 years, NEOLP: 45.7 ± 12.9 years, EOLP: 48.5 ± 14.5 years (summary statistics)	Both Genders
Kragelund 2019 [33]	Denmark	Randomized, double-blind, placebo-controlled, intervention, pilot study, longitudinal	22	NI	27–79 years	Both Genders (12 females and 10 males)
He 2021 [42]	China	Observational study	100	NI	Non-erosive group: average age 53.24 years (males: 53.84 years, females: 52.62 years); Erosive group: average age 52.98 years (males: 48.70 years, females: 55.99 years)	Both Genders (23 males and 36 females in the non-erosive group; 14 males and 27 females in the erosive group)
Liu 2018 [43]	Italy	Observational study using primary keratinocyte cultures from OLP patients, prospective design, single-site study	30	NI	37 to 88 years	23 females and 7 males
Molkenthin 2022 [44]	Germany	Retrospective, observational, cross-sectional, single-site study	268	NI	26–88 years	78.4% female and 21.6% male
Rezazadeh 2022 [45]	Iran	Cross-sectional observational study	40	32	Eligibility criteria: more than 18 years old; mean age of OLP patients: 48.83 (±9.34) years; mean age of control group: 40.21 (±10.32) years	Both Genders (OLP group: 22 females and 18 males; control group: 17 females and 15 males)
Li 2019 [46]	India	Observational study, cross-sectional, single-site	35	18	Healthy subjects: mean age 39.72 ± 11.02 years; Reticular OLP patients: mean age 43.58 ± 9.97 years; Erosive OLP patients: mean age 46.72 ± 9.80 years	Both Genders
Masaki 2011 [47]	Japan	Observational, controlled, single-site, prospective study design	15	7	Participants with OLP: 47–77 years. Healthy controls: 43–70 years	Both Genders (11 women and 4 men with OLP; 7 women in the control group)
He 2017 [48]	China	observational, cross-sectional, single-site, comparative study	43	21	BC group: mean age 53.6 ± 14.6 years; OLPE group: mean age 48.2 ± 15.5 years; OLPNE group: mean age 43.8 ± 14.1 years	Both Genders (more females than males)
Wang 2018 [49]	China	Controlled experimental laboratory study with primary cultured cells; not randomized, not blinded, single-site	5	0	24–41 years (actual participant ages); 18–50 years (eligibility criteria)	60% female and 40% male
Kumar 2014 [50]	India	Observational study with both prospective and retrospective components; single site; pathologists were blinded to clinical presentation and biopsy site	46	NI	Prospective group: 14–70 years; Retrospective group: 19–69 years	70% male and 30% female in prospective cases; 72% male and 28% female in retrospective cases
Tayebi 2023 [51]	Iran	Cross-sectional study	35	25	OLP patients: mean age 33.46 ± 13.41 years; healthy controls: mean age 31.85 ± 5.98 years	Both Genders (15 males, 20 females in OLP; 9 males and 11 females in healthy individuals)

**Table 2:** Extracted specific data included in the meta-analysis.

Authors	Risk of Bias	Total Sample		*C. albicans*		Non-*albicans Candida*		Oral Microbiome	
		OLP	Control	OLP	Control	OLP	Control	OLP	Control
Beibei, L. et al., 2024 [41]	Low Risk	49	10	0	0	4 *Candida* (dominant genus), *Saccharomycetes* *Aspergillaceae*, *Talaromyces*	0	49	10
Rezazadeh F. et al., 2022 [45]	Low Risk	40	32	40	29	Total sample with OLP found non-*albicans candida*: 0	Total sample control and healthy found non-*albicans candida*: 2 (1 *C. glabrata*, 1 *C. parapsilosis*)	40	27 *Candida albicans*, 1 case *C. glabrata*, 1 case *C. parapsilosis*
Li Y et al., 2019 [46]	Low Risk	35	18	34	17	4 Fungal species detected in OLP: *Candida*, *Aspergillus*, *Alternaria*, *Sclerotiniaceae*_unidentified	0	35	18
Masaki M., et al., 2011 [47]	Low Risk	15	7	15	7	4 The non-*Candida albicans* species found in the study were *C. glabrata*, *C. fukuyamaensis*, and *C. parapsilosis.*	0	12	2

**Table 3:** Comparison of effect sizes and heterogeneity with prior studies.

Study	Focus	OR (95% CI)	I^2^ (%)	Notes
Imabayashi et al., 2016 [52]	Oral mycobiome in OLP	Not reported (NGS-based)	NA	Descriptive shifts in fungal populations only
Bankvall et al., 2023 [31]	Oral microbiome in OLP (pilot)	Not reported (NGS-based)	NA	Reported altered taxa but no pooled quantitative effect
Rodriguez-Archilla & Fernandez-Torralbo, 2022 [18]	*C. albicans* in OLP	OR = 2.53 (1.39–4.59)	28%	Focused solely on *C. albicans*
**Present study (2025)**	*C. albicans*, non-*albicans*, oral microbiota	OR = 4.16 (1.28–13.51)	0%	First meta-analysis including broad mycobiome data

## Data Availability

The original contributions presented in this study are included in the article. Further inquiries can be directed to the corresponding author.
